# An Assessment of Clinically Important Differences on the Worst Pain Severity Item of the Modified Brief Pain Inventory in Patients with Diabetic Peripheral Neuropathic Pain

**DOI:** 10.1155/2018/2140420

**Published:** 2018-07-22

**Authors:** James Marcus, Kathryn Lasch, Yin Wan, Mei Yang, Ching Hsu, Domenico Merante

**Affiliations:** ^1^Pharmerit International, Bethesda, MD, USA; ^2^Pharmerit International, Boston, MA, USA; ^3^Daiichi Sankyo, Inc., Basking Ridge, NJ, USA; ^4^Daiichi Sankyo Development Ltd., Gerrards Cross, Buckinghamshire, UK

## Abstract

**Objectives:**

Using patient global impression of change (PGIC) as an anchor, an approximately 30% reduction on an 11-point numeric pain intensity rating scale (PI-NRS) is considered a clinically important difference (CID) in pain. Our objective was to define the CID for another pain measure, the worst pain severity (WPS) item of the modified Brief Pain Inventory (m-BPI).

**Methods:**

In this post hoc analysis of a double-blind, placebo-controlled, phase 2 study, 452 randomized patients with diabetic peripheral neuropathic pain (DPNP) were followed over 5 weeks, with m-BPI data collected weekly and PGIC at treatment conclusion. Receiver operating characteristic (ROC) curves (via logistic regression) were used to determine the changes in the m-BPI-WPS score that best predicted ordinal clinical improvement thresholds (i.e., “minimally improved” or better) on the PGIC.

**Results:**

Similar to the PI-NRS, a change of −3 (raw) or −33.3% from the baseline on the m-BPI-WPS optimized prediction for the “much improved” or better PGIC threshold and represents a CID. There was a high correspondence between observed and predicted PGIC categories at each PGIC threshold (ROC AUCs were 0.78–0.82).

**Conclusions:**

Worst pain on the m-BPI may be used to assess clinically important improvements in DPNP studies. Findings require validation in larger studies.

## 1. Introduction

Distal symmetric sensorimotor polyneuropathy, a significant complication of diabetes, is often associated with chronic neuropathic pain [1, 2]. Diabetic peripheral neuropathic pain (DPNP) affects approximately 50% of patients with diabetic neuropathy (16% of all diabetic patients) [3, 4] and has a substantial negative impact on patient functional status, work productivity, and quality of life [5–8]. Pain-related anxiety, depression, and sleep impairment and frequent comorbidity in patients with DPNP further exacerbate the patient burden [6–10].

Alleviation of pain is the cornerstone of patient management. A number of medications are approved or recommended for treatment of DPNP [11–16]; however, pain relief is elusive because of issues of suboptimal effectiveness or tolerability [17, 18]. Although simple analgesics provide partial, short-term relief, sustained control of neuropathic pain requires therapies that are are more specifically targeted, better tolerated, and more effective over time [19].

Demonstrated effectiveness in terms of pain reduction, assessed using a patient-reported outcomes (PRO) measure, is essential for the approval of new pain treatments. However, the interpretability of an improvement in pain scores and whether they are truly meaningful and clinically relevant is equally important [20]. The emphasis on patient-centered care is highlighted in guidance issued by the US Food and Drug Administration (FDA) outlining the psychometric attributes (reliability, validity, and clinically meaningful score changes) that should be considered in the development of a PRO measure [21].

Pain rating scales, assessed using several different instruments, are widely used PRO measures [22]. In studies of chronic pain conditions, an 11-point pain intensity numeric rating scale (PI-NRS), ranging from 0 = no pain to 10 = worst possible pain, is the gold standard [20]. Farrar et al. [20] conducted an oft-cited study of the clinically important difference (CID) in pain improvement in which each morning, before taking study medication, the patients were asked to circle the number that best described their pain over the preceding 24 hours. An average daily pain score (ADPS) was calculated based on the responses. Farrar et al. recognized the importance of defining the level of changes on the PI-NRS that best reflects what patients consider to be a clinically important improvement. Data from 10 studies of pregabalin for the treatment of various chronic pain conditions (*N* = 2879) were used to determine numeric changes in the PI-NRS (e.g., −1 or −2) that were most closely associated with an improvement on the patient global impression of change (PGIC), a commonly used validated measure of patient global self-assessment of the health status [23]. The results suggested that a reduction of approximately 2 points on the PI-NRS (or 30% change) represented a CID.

The PI-NRS does not qualify the patient pain experience beyond its anchors 0 = “no pain” and 11 = “worst possible pain.” Pain thresholds may differ among patients, and their largely subjective interpretation of the measurement scale may lead them to report on different facets of pain (e.g., average pain or worst pain) when responding to the PI-NRS―limiting the intrinsic meaning of its associated CID [24]. The Brief Pain Inventory (BPI), originally developed for assessment of cancer pain, is another commonly used PRO measure in chronic pain studies [25]. While the PI-NRS is typically used to assess average pain over the last 24 hours, the BPI characterizes an additional 3 dimensions of pain intensity (pain at its worst in the last 24 hours, pain at its least in the last 24 hours, and pain right now) measured using an 11-point NRS ranging from 0 = “no pain” to 10 = “pain as bad as you can imagine.” By distinguishing between different types of pain, the BPI increases the likelihood that different patients will interpret a given question in a similar way. The BPI has been modified for use in other pain conditions, validated in numerous pain studies [26–32], and Farrar et al. [33] have defined a CID (change of 34%) on the worst pain item of the BPI based on data from duloxetine clinical trials of patients with DPNP and fibromyalgia. Since CID can vary depending on patient population and clinical context [34], it is important to show similar results for multiple pain indications.

The worst pain severity item of the BPI (BPI-WPS) has consistently demonstrated the highest reliability (internal consistency) across the BPI validation studies, and the psychometric properties of the worst pain item meet the standards set forth in the FDA guidance for PRO measures [35]. Moreover, in a recently issued draft guidance, the FDA recommended the use of an instrument that assesses worst pain over a relatively short period (no longer than 24 hours) to measure the primary efficacy endpoint in clinical trials [36], making the BPI-WPS an optimal candidate for use in pivotal studies of chronic pain treatment.

In this post hoc analysis, our main objective was to evaluate the association of the worst pain severity item of the BPI, modified for use in patients with DPNP (m-BPI-WPS) [30], with improvements on the PGIC and to quantify numeric changes in worst pain scores that constitute a CID. A secondary objective was to evaluate the association of the worst and average pain severity items of the m-BPI with the ADPS derived from the standard PI-NRS.

## 2. Materials and Methods

### 2.1. Patients and Study Design

This post hoc analysis was based on data from a randomized, double-blind, placebo-controlled, active comparator-controlled, adaptive, proof-of-concept, phase 2 study of the efficacy and safety of mirogabalin monobenzenesulfonate (DS-5565, Daiichi Sankyo Co., Ltd., Tokyo, Japan, herein referred to as mirogabalin) for the treatment of DPNP (Clinicaltrials.gov identifier NCT01496365) [37–39]. The adaptive trial design enabled efficient determination of the optimal dosing for safety, while reducing safety risks for patients. A total of 452 adults with type 1 or 2 diabetes who met the study eligibility criteria were randomly assigned (the 2 : 1 : 1 : 1 : 1 : 1 : 1 ratio) to 1 of 7 treatment groups: placebo, dose-ranging mirogabalin (5, 10, 15, 20, and 30 mg/day), or pregabalin (300 mg/day) for 5 weeks. The study duration comprised approximately 9 weeks, reflecting an approximate 3-week screening/baseline period, a 5-week treatment period, and a 1-week follow-up period after the last dose of study medication or the end-of-treatment visit.

This study was conducted in accordance with the Declaration of Helsinki, the International Conference on Harmonisation (ICH) consolidated Guideline E6 for Good Clinical Practice, and all other applicable regulatory requirements. All patients provided written informed consent prior to participating in the study.

### 2.2. Measures

The study's primary efficacy measure was the change in the pain score from the baseline to week 5 or the end of study measured using the ADPS on the 11-point PI-NRS (0 = “no pain” to 10 = “worst possible pain”). The ADPS was calculated as the mean of the last 7 entries in the patients' daily diaries prior to randomization (baseline) and the last 7 entries while taking study medication (endpoint). Weekly change in ADPS was included as a secondary efficacy measure.

The m-BPI, the focus of this paper, was also included in the study as a secondary efficacy measure: its 4-item pain severity scale (pain at its worst in the past 24 hours, pain at its least in the past 24 hours, pain on the average, and pain right now) was assessed weekly from randomization through the end of treatment. A PGIC was assessed at the end of treatment on a 7-point PGIC categorical scale: “Since the start of the study, my overall status is …” 1 = very much improved, 2 = much improved, 3 = minimally improved, 4 = no change, 5 = minimally worse, 6 = much worse, and 7 = very much worse.

### 2.3. Statistical Analyses

The SAS software system (PC version 9.4, SAS Institute Inc., Cary, NC) was used to complete all data analyses. Following the methodology outlined by Farrar et al. [20], ordinal logistic regression analyses were used to evaluate the relationship between the worst pain (m-BPI-WPS) item and the PGIC. PGIC categories served as the dependent variable, and either the raw or the percentage change in m-BPI-WPS scores served as the independent variable.

The ordinal logistic regression, using raw or percent change in worst pain as a predictor, compares the cumulative odds of appearing in a given PGIC category or better: “very much improved” (i.e., PGIC scale 1 versus 2–7), “much improved” or better (i.e., PGIC scales 1–2 versus 3–7), and “minimally improved” or better (i.e., PGIC scales 1–3 versus 4–7). The predicted probabilities of appearing on a given side of discretized PGIC categories are compared against a range of cutoff thresholds to construct receiver operating characteristic (ROC) curves that plot the rate of correct predictions (observed PGIC matches prediction; sensitivity) versus false alarms (predicted to be in the higher category, but actually observed in the lower category; 1−specificity). The area under the ROC curve (AUC), reported as the *c* statistic from the logistic regression, represents the total overall association between the m-BPI-WPS score and the discretized PGIC category used to construct the specific curve (AUC/*c* is bounded from 0.50 to 1.00, where a value of 0.50 (i.e., the ROC diagonal) would indicate that worst pain has no ability to predict PGIC). Assuming equal importance of sensitivity and specificity, the probability cutoff that maximizes prediction using change in worst pain is located at the point at which sensitivity and specificity are the closest to being equal; this occurs at the intersection of a 45° tangent line with the ROC curve (the steepest rate of change) [20]. The probability cutoff that resulted in this intersection (i.e., point of sensitivity/specificity equality) can be recovered and compared to the predicted probabilities at each change score to find the change score with the closest match between its predicted probability and the optimal cutoff. In addition, the raw change of the m-BPI-WPS score was graphically displayed by PGIC categories using a box plot.

To address our secondary objective, polyserial correlations were used to understand the relationships among the various items of the m-BPI pain severity scale and the ADPS at study endpoint (week 5). Polyserial correlations are appropriate when examining the relationship between continuous (ADPS) and ordinal variables (individual items of the m-BPI) when it is assumed that the ordinal variable has an underlying continuous dimension [40]. A scale proposed by Chung [41] was used to describe the strength of the correlation coefficients, specifically 0.8 to 1.0 (very strong relationship), 0.6 to 0.8 (strong relationship), 0.4 to 0.6 (moderate relationship), 0.2 to 0.4 (weak relationship), and 0.0 to 0.2 (weak or no relationship). For items that had a strong correlation with the ADPS, regression analyses were performed to better understand the direction of the correlation (i.e., the slope of the relationship) with the ADPS at week 5 as the dependent variable and the individual items of the m-BPI as independent variables.

## 3. Results

### 3.1. ROC Curves for PGIC and Changes in the m-BPI-WPS Scores

**Figure 1:**
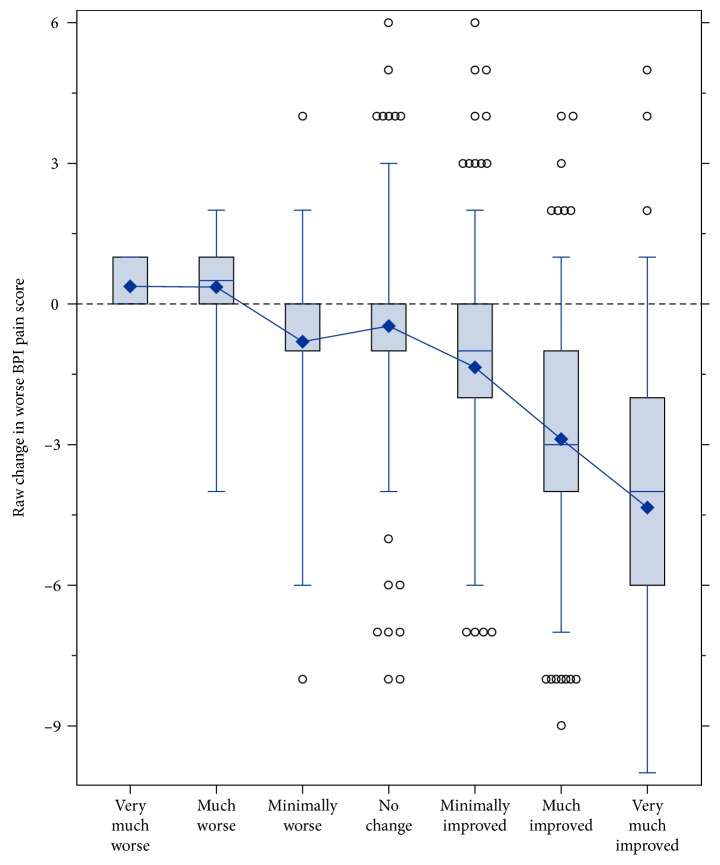
Box plot of raw change in the m-BPI score from the baseline to week 5/end of study by PGIC categories. The center line inside the box represents the median, the box's hinges are the 25th and 75th percentile, the whiskers bound the central 95 percent of the distribution, the circles beyond the whiskers are outliers, and the diamond represents the mean. BPI, Brief Pain Inventory; m-BPI, modified Brief Pain Inventory; PGIC, patient global impression of change.

A total of 424 patients had nonmissing PGIC data. The box plot in Figure 1 shows the full distribution of the change in the m-BPI-WPS score for each PGIC category. This figure illustrates that almost all patients who considered themselves “minimally improved or better” (73%), “much improved or better” (44%), or “very much improved” (13%) had at least some decrease in the m-BPI-WPS score, and most of the patients had a decrease of 2 points or more.

Via ordinal logistic regression, a change in the m-BPI-WPS score was an effective predictor of the cumulative PGIC category, satisfying the proportional odds assumption *χ*^2^(2) = 2.49, *p*=0.29, and achieving a proportional reduction in the error in predicting PGIC of *R*^2^_Nag_ = 0.37. Each 1-point reduction in worst pain increased the odds of advancing to a higher PGIC category by 1.69 (95% confidence interval: 1.55, 1.84).

While the m-BPI has 3 other pain measures—least, average, and pain now—they are not discussed further in the manuscript, either as separately or multivariately modeled predictors. Beyond the reasons cited at the end of Introduction for the primacy of worst pain, empirical evidence was collected for its primacy as well. There was compelling evidence for multicollinearity, with the correlations between the measures at the end of treatment ranging from 0.77 to 0.90. Additionally, when all 4 were included in the model, the 3 others regressed toward 0, and worst pain remained the dominant predictor when it was paired with any 1 or 2 of the other pain measures (models without it had average pain take its place, with a similar regression coefficient). When fitting each pain as the sole predictor in separate models, their regression coefficients were not significantly different (*β* = 0.47–0.56, SE[*β*] = 0.05), indicating little benefit to exploring them further.

**Figure 2:**
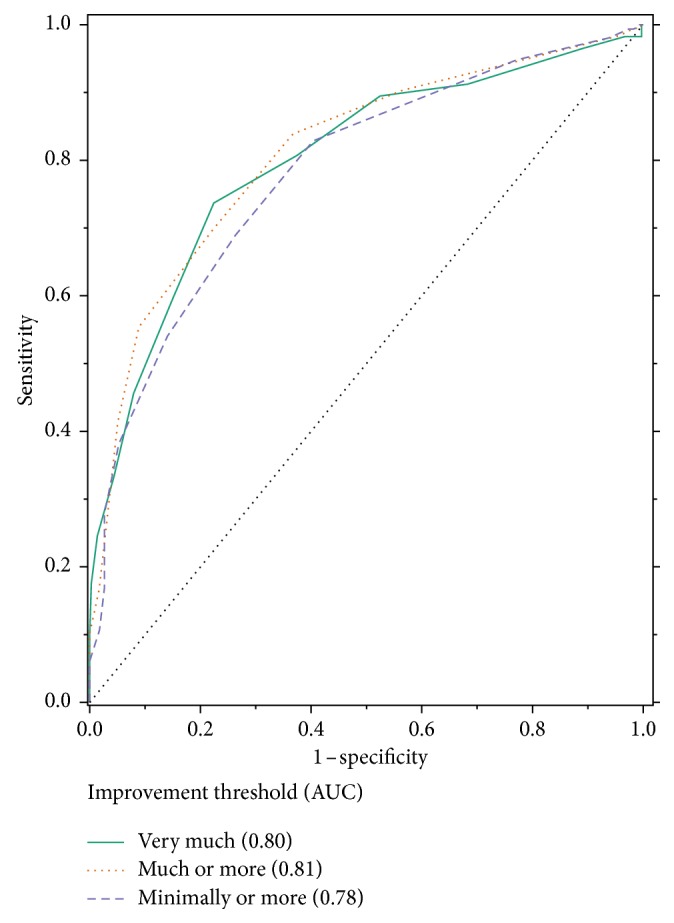
ROC curve of raw change in the m-BPI-WPS score from the baseline to week 5/end of the study and PGIC. AUC, area under the curve; m-BPI-WPS, modified Brief Pain Inventory-worst pain severity; ROC, receiver operating characteristic.

**Figure 3:**
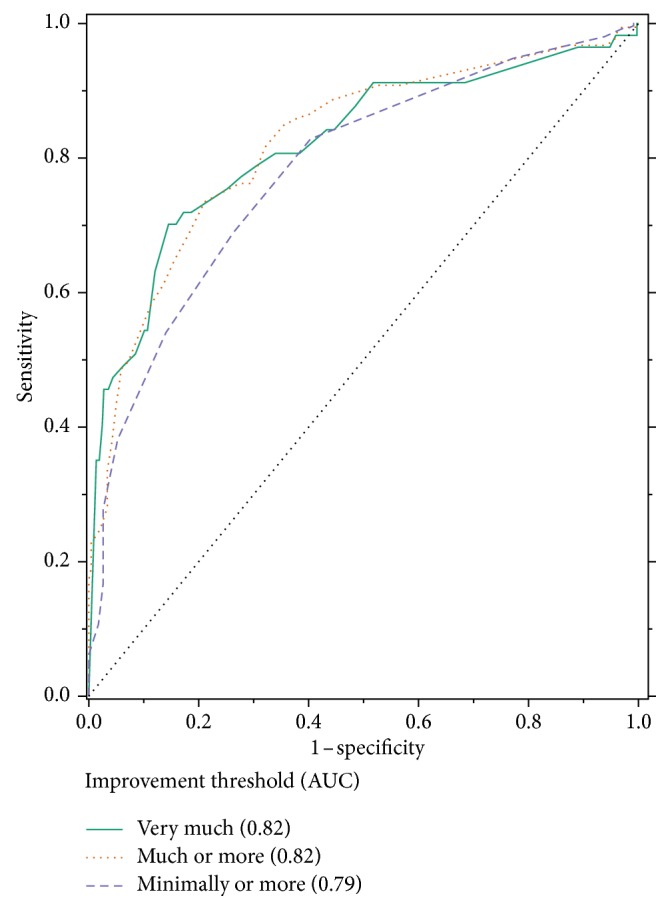
ROC curve of percentage change in the m-BPI-WPS score from the baseline to week 5/end of the study and PGIC. AUC, area under the curve; m-BPI-WPS, modified Brief Pain Inventory-worst pain severity; PGIC, patient global impression of change; ROC, receiver operating characteristic.

Table 1 provides specific values generated from the ROC analyses for both raw change and percentage change in the m-BPI-WPS score best associated with several definitions of clinically important improvement (i.e., “minimally improved” or better, “much improved” or better, and “very much improved” only). The areas under the ROC curves for the m-BPI-WPS raw score change and percentage change (Figures 2 and 3) are nearly identical for each definition of improvement. A raw change of −3 (70.3% sensitivity and 77.8% specificity) and a percentage change of −33.3% (76.2% sensitivity and 72.8% specificity) were best associated with the PGIC category “much or very much improved” (Table 1).

While not the focus of the paper, we also assessed the impact of the study's treatment conditions using an ordinal logistic model that fit the PGIC cumulative category as a function of change in worst pain score, treatment, and their interaction. Overall model fit improved slightly, with the proportional reduction in the error in predicting the PGIC increasing from *R*^2^_Nag_ = 0.37 to *R*^2^_Nag_ = 0.40 and global AUC slightly increasing from *c* = 0.77 to *c* = 0.78. There was no statistically significant interaction between worst pain and treatment, *F*(6407) = 1.20, *p*=0.30, but there were statistically significant main effects for both worst pain, *F*(1407) =133.91, *p* < 0.0001, and for treatment, *F*(6407) = 2.62, *p*=0.02. For worst pain, the odds ratio improved from 1.69 to 1.87. As the treatment conditions were largely aimed at examining dose response of mirogabalin, the main effect of treatment was analyzed via Helmert contrasts. Compared with a placebo, mirogabalin doses ≥5 mg increased the odds of advancing PGIC categories by 1.63, *z* = 3.18, *p*_Holm-Sidak_ = 0.01; there was no significant difference in the odds when comparing 5 mg to higher doses. In addition, model results were very similar when worst pain was expressed as the percent change instead of the raw change.

### 3.2. Correlation Analysis

The ADPS was highly correlated (i.e., very strong relationships) with all the items of the m-BPI pain severity scale, including “pain at its worst in the past 24 hours,” “pain at its least in the past 24 hours,” “pain on the average,” and “pain right now.” The correlation coefficient was the highest for the worst pain item (0.87) and lowest for the least pain item (0.81). The correlations and regression slopes are presented in Table 2. The regression slopes represent the unit change in the ADPS associated with every 1-point change in the predictor variables (individual items of the m-BPI pain severity scale). Consistent with the correlation analysis, the regression slopes indicate that all items of the m-BPI had a significant association with the ADPS, with the association being the highest for the average pain item (slope = 0.89) and lowest for the pain right now item (slope = 0.80).

## 4. Discussion

This post hoc analysis demonstrates that the m-BPI-WPS is closely associated with the PGIC and may be used to describe clinically meaningful changes in patient assessment of DPNP. The results also suggest that a 3-point or 33.3% reduction in the m-BPI-WPS score represents a clinically important difference. Our findings are consistent with previous research [20] and reiterate the CID on the BPI worst, least, and average pain severity scales established by Farrar et al. [33]. Although the Farrar et al. study established CID based on associations of the BPI items with patient-perceived improvements at endpoint as measured by the 7-point patient global impression of improvement scale, our finding of a 33.3% reduction representing a CID is almost identical to the 34% reduction reported in that study [33]. Our analyses also describe changes in m-BPI-WPS scores associated with the various categories of global improvement, information that could potentially be useful in evaluating the relative effectiveness of chronic pain treatments in clinical practice. For example, a 50% reduction in the m-BPI-WPS score represents the highest level of clinical improvement, whereas a 20% reduction indicates minimal improvement. These benchmarks could be useful to clinicians in guiding prescribing decisions for individual patients.

Although the FDA recently recommended the use of worst pain as a primary endpoint in pain clinical studies [36], the ADPS has historically been used as the gold standard PRO measure. Therefore, to facilitate comparison of our findings with those of previous studies, we wanted to investigate if the m-BPI-WPS item correlated with the ADPS. Our results in DPNP patients indicated a strong correlation of all items of the m-BPI pain severity scale with the ADPS, with the correlations being the strongest for the worst and average pain items. Accordingly, our results suggest that the use of the worst pain score may improve the interpretability of pain measures results in DPNP clinical trials.

Our analyses are subject to several limitations. Data for our study were derived from a single phase 2 study. Therefore, in addition to a small sample size, the homogeneity of the study sample (owing to specific patient recruitment criteria) restricts the generalizability of our findings. It is possible that the CID established in our study may not be relevant for DPNP patients in usual care who present with multiple comorbidities or a disease profile different from those of our trial patients. Although the association of the PGIC with pain scores suggests that a patient's pain experience is closely related to his/her evaluation of the health status, it is possible that patients with more debilitating comorbidities may report global health improvements that are inconsistent with pain reduction.

Our results are based on patients with DPNP who presented with moderate to severe ADPS scores at the baseline (mean ADPS was 7.0 in the placebo arm, 6.7 across all 5 mirogabalin treatment arms, and 6.6 in the pregabalin arm). To facilitate application of our findings in clinical practice, the analysis presented here should be repeated with data collected through observational studies and should include a more representative patient population. In addition, our study sample comprised patients with DPNP, and findings should be applied with caution to patients with other chronic pain conditions.

Finally, the 5-week duration of the treatment period of this clinical trial may not have been long enough to observe possible changes over time in the correspondence between the m-BPI-WPS and the PGIC. However, Farrar et al. showed that a different PRO (the PI-NRS) corresponded well with PGIC regardless of the study length [33]. Therefore, a longer study duration is not expected to impact these results.

## 5. Conclusions

Our results in DPNP patients present preliminary values for raw and percentage changes in scores of the m-BPI-WPS that constitute a CID. Although the generalizability of our findings is limited owing to the small sample size of this adaptive and innovative phase 2 study in DPNP patients, our results not only reinforce previous findings but also add external validity. Researchers have suggested that using a standard definition of CID in studies of chronic pain treatment will simplify comparisons of treatment effects. Our findings provide additional data to support the establishment of such a universal definition of CID.

## Figures and Tables

**Table 1:** ROC analyses: model statistics at a tangent for the change in the m-BPI-WPS score.

Pain score change (type)	PGIC	AUC	Sensitivity (%)	Specificity (%)	Value (change in the pain score)^*∗*^	Total accuracy (%)
Raw change	Very much improved	0.801	73.7	77.7	−4	77.1
Raw change	Much or very much improved	0.814	70.3	77.8	−3	74.5
Raw change	Minimally, much, or very much improved	0.784	69.2	74.1	−2	70.5
Percentage change^∗^	Very much improved	0.820	75.4	74.9	−50.0	75.0
Percentage change^∗^	Much or very much improved	0.823	76.2	72.8	−33.3	74.3
Percentage change^∗^	Minimally, much, or very much improved	0.790	72.7	73.3	−20.0	72.9

Percentage change = raw change in the BPI worst pain score/baseline pain score. ^*∗*^The value of change in the pain score is defined by the intersection of a 45° tangent line with each ROC curve, which is mathematically equivalent to choosing the point at which sensitivity and specificity are the closest to being equal. AUC, area under the curve; m-BPI-WPS, modified Brief Pain Inventory-worst pain severity; PGIC, patient global impression of change; ROC, receiver operating characteristic.

**Table 2:** Polyserial correlations and regression slopes of the m-BPI pain severity scale items with ADPS.

m-BPI pain severity scale	*n*	Correlation coefficient	Slope of items as a predictor for ADPS	*p* value
Pain right now	385	0.834	0.800	<0.001
Pain at its least in the last 24 hours	385	0.811	0.845	<0.001
Pain at its worst in the last 24 hours	385	0.874	0.820	<0.001
Pain on the average	384	0.842	0.890	<0.001

Patients with missing data on the BPI pain scales or ADPS at the endpoint were excluded from the correlation analysis. ADPS, average daily pain score; BPI, Brief Pain Inventory; m-BPI, modified Brief Pain Inventory.

## Data Availability

Supporting data for this post hoc analysis can be found in the following publication which is cited as [37].
